# Implementation costs of hospital-based computerised decision support systems: a systematic review

**DOI:** 10.1186/s13012-023-01261-8

**Published:** 2023-02-24

**Authors:** Thomasina Donovan, Bridget Abell, Manasha Fernando, Steven M. McPhail, Hannah E. Carter

**Affiliations:** 1 Australian Centre for Health Services Innovation and Centre for Healthcare Transformation, School of Public Health and Social Work, Faculty of Health, Queensland University of Technology, Brisbane, QLD Australia; 2 Digital Health and Informatics, Metro South Health, Brisbane, QLD Australia

**Keywords:** Implementation costs, Computerised decision support systems, CDSS, Hospital, Digital health, Economic evaluation

## Abstract

**Background:**

The importance of accurately costing implementation strategies is increasingly recognised within the field of implementation science. However, there is a lack of methodological guidance for costing implementation, particularly within digital health settings. This study reports on a systematic review of costing analyses conducted alongside implementation of hospital-based computerised decision support systems.

**Methods:**

PubMed, Embase, Scopus and CINAHL databases were searched between January 2010 and August 2021. Two reviewers independently screened and selected original research studies that were conducted in a hospital setting, examined the implementation of a computerised decision support systems and reported implementation costs. The Expert Recommendations for Implementing Change Framework was used to identify and categorise implementation strategies into clusters. A previously published costing framework was applied to describe the methods used to measure and value implementation costs. The reporting quality of included studies was assessed using the Consolidated Health Economic Evaluation Reporting Standards checklist.

**Results:**

Titles and abstracts of 1836 articles were screened, with nine articles eligible for inclusion in the review. Implementation costs were most frequently reported under the ‘evaluative and iterative strategies’ cluster, followed by ‘provide interactive assistance’. Labour was the largest implementation-related cost in the included papers, irrespective of implementation strategy. Other reported costs included consumables, durable assets and physical space, which was mostly associated with stakeholder training. The methods used to cost implementation were often unclear. There was variation across studies in the overall quality of reporting.

**Conclusions:**

A relatively small number of papers have described computerised decision support systems implementation costs, and the methods used to measure and value these costs were not well reported. Priorities for future research should include establishing consistent terminology and appropriate methods for estimating and reporting on implementation costs.

**Trial registration:**

The review protocol is registered with PROSPERO (ID: CRD42021272948).

**Supplementary Information:**

The online version contains supplementary material available at 10.1186/s13012-023-01261-8.

Contributions to the literature

- A low number of papers have reported the costs associated with implementing computerised decision support systems in hospitals. The methods used to cost implementation strategies were not well reported and complicated by inconsistent terminology.
- Economic evaluations should include implementation costs to prevent an underestimation of costs. However, there is a lack of methodological support to cost implementation strategies, particularly within the context of digital health.
- This review highlighted the importance of establishing a consistent terminology and appropriate methods for estimating and reporting on implementation costs to account for technology lifecycles (including de-implementation costs) and workforce productivity impacts.

## Introduction

Computerised decision support systems (CDSS) are digital health technologies designed to analyse patient’s clinical data to assist in clinical decision-making at the point of care [1]. CDSS can be knowledge based which involves predetermined rules based on literature, clinical practice or patients, or non-knowledge based which utilises advanced statistical pattern recognition, including artificial intelligence and machine learning, to produce an action or output from the data [2]. The use of CDSS, and other digital health innovations, is increasing as hospitals move towards fully electronic systems for capturing and communicating patient data. However, the complexity of health systems presents challenges for CDSS implementation [3]. Commonly reported barriers include the costs of implementing new systems, as well as technology-specific concerns [4]. Changes to workflow practices associated with the adoption of new digital health initiatives often requires behavioural change from healthcare workers to ensure implementation is successful and sustainable [5].

Implementation science provides a method to understand the factors that support the successful adoption of evidenced-based innovations. Implementation strategies can then be used to target these factors from an innovation, individual, organisational or system perspective. In evaluating implementation, cost is understood to be a necessary element for success and sustainability [6]. However, costs associated with implementation strategies are under-reported in the literature and often excluded from economic evaluations despite being a recognised implementation outcome [7–9].

A lack of common language surrounding the term ‘implementation’ across disciplines can lead to ambiguity about what constitutes an implementation cost. In the implementation science literature, ‘implementation’ refers to the strategy of actions/methods used to facilitate behaviour change to support adoption, integration and sustainment of innovations into clinical practice [10]. Conversely, in an IT context ‘implementation’ more commonly refers to the operationalisation of an innovation [11], while in software engineering, ‘implementation’ often includes the software coding process [12]. This review aligns with the implementation science definition and is concerned with the costs associated with implementation strategies.

Approaches to costing implementation strategies are emerging; however, none have been designed for the unique context of digital health innovations. Technological considerations are inter-related with other social and organisational considerations, and unintended consequences can emerge if these dimensions are not balanced, which can lead to implementation failure [13]. An enhanced understanding of the resourcing requirements associated with the implementation of digital health initiatives would provide decision-makers with more accurate and transparent information that would likely support successful implementation efforts.

The aim of this review was to describe the nature of implementation costs that have been estimated within hospital-based CDSS initiatives and to document the methods that have been used to measure and value these costs. This review may inform ongoing research efforts to establish appropriate methodology for costing implementation efforts within digital health and healthcare settings more broadly.

## Methods

This systematic review is reported according to the Preferred Reporting Items for Systematic Reviews and Meta-Analyses (PRISMA) guidelines for systemic reviews [14] and registered with PROSPERO (ID: CRD42021272948).

### Eligibility criteria

The population, intervention, comparator, outcomes, study design (PICOS) framework [15] was used to define the eligibility criteria. Studies that met the following criteria were included:

- Population—Conducted in a hospital setting, encompassing both inpatient (tertiary) and outpatient (secondary) services.
- Intervention/exposure—CDSS defined as a computerised system used by healthcare professionals to assist in clinical decision-making at point of care [2]. This excludes CDSS's that do not involve a decision support element including technologies that are only concerned with medical records, communication, information exchange, clinical performance, infection outbreak warning and shared decision-making.
- Outcomes—Reported the direct and indirect costs of implementation strategies associated with the implementation of CDSS.
- Study design—Studies reporting original research.

Articles were excluded if as follows: published prior to 2010 (for pragmatic reasons and due to rapidly developing literature in this field); they were an abstract or dissertation; the setting was primary care, a virtual hospital, hospital in the home, other in home services, or not specified as hospital; they reported on CDSS development but not implementation; they reported on a digital health intervention that did not involve a decision support element (consistent with our definition of CDSS); or no implementation strategies were costed. No language restrictions were applied.

### Search strategy

PubMed, Embase (Elsevier), Scopus (Elsevier) and CINAHL (EBSCOhost) databases were searched using a combination of subject headings and keywords across four categories: ‘cost’, ‘implementation’, ‘CDSS’ and ‘hospital’. The search results were restricted to journal articles published between January 2010 and 02 August 2021 (last date searched). Forward and backward citation searching was performed on included papers. The detailed search strategy is outlined in Additional file 1.

### Study selection

All records retrieved from the search were imported into EndNote, deduplicated and then imported into Rayyan for screening [16]. Two authors (T. D. and M. F.) independently performed title and abstract screening of all records in Rayyan. Abstracts that met the inclusion criteria were progressed to full-text screening which was independently conducted by two authors (T. D. and H. C.). Discrepancies at any stage in the screening process were resolved through discussion with T. D., M. F. and H. C. to reach consensus on which articles to include. Study investigators were not contacted for more information.

### Data collection and analysis

One author (T. D.) extracted the data from the included studies using a form that was piloted and revised by T. D. and H. C. The data extraction form included study characteristics (author, year published, country, study design) as well as the clinical setting, clinical condition, CDSS type, implementation science theory/model/framework employed [17], economic study design [18], implementation strategies used and implementation costs.

The Expert Recommendations for Implementing Change (ERIC) framework was used to identify implementation strategies and related CDSS implementation costs. Implementation strategies reported in the included papers were mapped to one or more discrete implementation strategies defined in the ERIC framework. These individual strategies were then categorised into one of nine clusters of implementation strategies [19]. The ‘humans, “things” and space’ costing framework was applied to identify and describe the methods used to measure CDSS implementation costs. The framework costs hospital interventions by capturing the staff time of ‘humans’ involved, ‘things’ including durable assets and consumables and the physical space utilised [20].

### Data synthesis

Data were collated and synthesised using narrative and descriptive summaries. No attempt at meta-analysis was made given the heterogeneity in target population, intervention, study design and outcome measures across included studies.

### Quality assessment

The reporting quality of included papers was assessed using the Consolidated Health Economic Evaluation Reporting Standards (CHEERS) checklist because most were economic evaluations [21]. Two authors (T. D. and H. C.) independently conducted the quality assessment, and discrepancies were resolved through discussion.

## Results

### Study selection

**Fig. 1:**
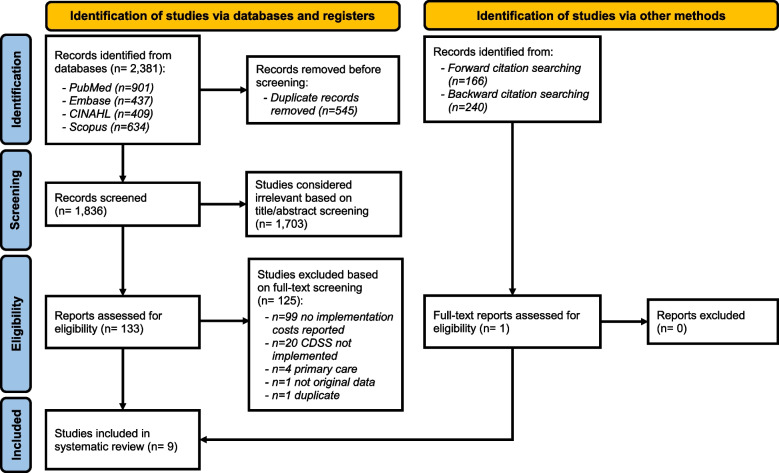
PRISMA flowchart of study selection process. Study selection was in accordance with the Preferred Reporting Items for Systematic Reviews and Meta-Analyses (PRISMA) guidelines for systemic reviews [14], and the flowchart is sourced from [22]

Figure 1 illustrates the article selection process. The search strategy yielded 1836 articles after removing duplicates, from which we reviewed the full text of 133 articles against the defined inclusion criteria. We excluded 125 of these for the following reasons: was a duplicate (*n* = 1); not an original study (*n* = 1); primary care setting (*n* = 4); CDSS was developed but not implemented (*n* = 20); or CDSS implementation was not costed (*n* = 99). A total of 8 remaining articles were included in the review [23–30]. Forward citation searching (*n* = 166) and backward citation searching (*n* = 240) were conducted on the 8 included papers resulting in the identification of 1 eligible paper that subsequently met the inclusion criteria [31]. Therefore, 9 papers were included in this review.

### Study characteristics

Key characteristics of the included studies are summarised in Table 1. All except one of the nine included studies were from high-income countries. Two included papers conducted prospective studies across multiple centres [30, 31]. All other studies were retrospective observational single-centre designs that described implementation within a single service [23–29].

**Table 1:** Characteristics of included papers

First author, year (reference)	Country	Study design	Clinical setting	Clinical condition	CDSS type	Implementation science theory/model/framework	Economic study design
**Afshar, 2019 [**23**]**	USA	Single centre, observational, retrospective	Emergency department	Preventing in-hospital sepsis	Order sets and modified early warning system (MEWS)	Not applied	Cost-effectiveness analysis
**Agulnik, 2019 [**24**]**	Guatemala	Single centre, observational, retrospective,	National Paediatric Oncology Unit	Early identification of deterioration	Paediatric early warning systems (PEWS)	Not applied	Cost-benefit analysis
**Castellanos, 2013 [**25**]**	Germany	Single centre, observational, retrospective	Interdisciplinary surgical intensive care unit	Bedside clinical document	The patient data management systems (PDMS)	Not applied	Cost analysis
**Field, 2012 [**26**]**	USA	Single centre, observational, retrospective	Ambulatory care	Transitional care	Automated alert	Not applied	Cost analysis
**Forrester, 2014 [**27**]**	USA	Single centre, observational, retrospective	Ambulatory care	Reduce medication errors and adverse drug events (ADE)	Computerised provider order entry (CPOE)	Not applied	Cost-effectiveness analysis
**Swart, 2020 [**28**]**	UK	Single centre, observational, retrospective	Haematology department	Prevent inappropriate use of blood and blood components	Patient blood management (PBM)	Not applied	Cost analysis
**Vermeulen, 2014 [**31**]**	Netherlands	Multi-centre, interrupted time series, prospectively	General internal, geriatric and gastroenterology wards	Reduce medication errors and ADE	CPOE with a basic CDSS	Not applied	Cost-effectiveness analysis
**Westbrook, 2015 [**29**]**	Australia	Single centre, observational, retrospective	Cardiology ward	Reduce medication errors and ADE	Electronic medication management system (eMMS)	Not applied	Cost-effectiveness analysis
**Zimlichman, 2013 [**30**]**	USA	Multi-centre, observational, prospective before-and-after	Admitting services hospital wide (excluding psychiatric and neonatal services)	Reduce medication errors and ADE	CPOE	Not applied	Cost-benefit analysis

All articles were conducted within the context of a health economic study design. Four of the studies were cost-effectiveness analyses [23, 27, 29, 31], three were cost analyses [25, 26, 28] and two were cost-benefit analyses [24, 30]. Economic evaluations took the perspective of the health service [23, 27–29, 31] or was not specified [24–26, 30]. None of the articles reported using an implementation science theory, model or framework to assist CDSS implementation.

### CDSS characteristics

The type of CDSS implemented varied across the included studies. Three papers included an alert or early warning system which aided in the identification of patients at risk according to the respective clinical guideline [23, 24, 26]. For example, one of the papers investigated a paediatric early warning system that used a multicomponent scoring tool with an action algorithm to identify hospitalised children with clinical deterioration [24]. Three papers implemented a computerised provider order entry (CPOE) system which aims to reduce medication errors and adverse drug events by minimising illegible writing, unstructured orders and dosing variability [27, 30, 31]. Another study implemented order sets which are a bundle of clinical orders grouped together to improve adherence to clinical guidelines [23]. Three papers implemented a management system for specific clinical contexts. Management systems are multi-functional, typically multidisciplinary and often integrate CPOE systems. A patient data management system was investigated in an included paper which supported bedside clinical documentation. Its functionalities included data acquisition from monitoring and medical devices, bedside CPOE facilitating calculations of drug doses and fluid balances, import interfaces from laboratory/microbiology/radiology data and surgery reports, automated calculation of ventilation times, automated scoring and semiautomated coding of diagnoses/procedures with interface for exporting data directly to the electronic billing system [25]. Another included study implemented a patient blood management system which was defined as, "an evidence-based, multidisciplinary approach to optimising the care of patients who might need transfusion". It aimed to ensure optimal treatment was given while reducing avoidable/inappropriate use of blood and blood components [28]. Electronic medication management system (eMMS) was the final management system studied in an included paper. The eMMS interfaced with the existing CPOE allowing doctors to prescribe medication electronically as well as having functionalities to alert for drug allergy checking, pregnancy warnings, therapeutic duplication and some dose-range checking [29].

### CDSS implementation strategies and cost

**Fig. 2:**
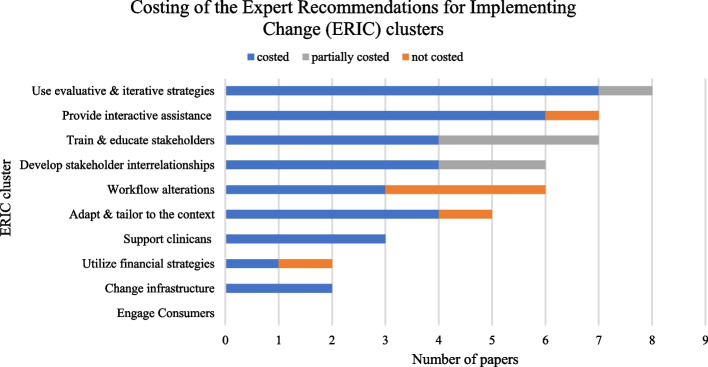
Costing of the Expert Recommendations for Implementing Change (ERIC) clusters in the included papers. The figure illustrates the number of included papers that reported a discrete implementation strategy categorised to an ERIC implementation [19]. Within individual papers, each ERIC cluster was determined to be ‘costed’, ‘partially costed’ or ‘not costed’

Implementation strategies were extracted from the information contained in the included papers and categorised against the ERIC framework. Identified implementation strategies were then cross-checked with reported costs in the included papers to determine if the strategy had been costed. A summary of these findings is presented in Table 2. Figure 2 provides a high-level illustration on the number of papers that costed, partially costed or did not report costs for each cluster of implementation strategies.

**Table 2:** Implementation strategy categorisation and determination of whether it was costed in the included paper

ERIC cluster and strategy		Implementation strategy in included paper	Citation/s	Costed
Use evaluative & iterative strategies	Assess for readiness and identify barriers and facilitators	A multidisciplinary team of key informants at National Paediatric Oncology (UNOP) established paediatric early warning system (PEWS) implementation was feasible based on long-term institutional dedication to quality improvement, strong nursing buy-in for the project and paediatric intensive care unit (ICU) commitment to improving outreach and monitoring in the hospital wards	(From former paper [32]) [24]	N
		Before the implementation, the current situation (organisational aspects of the medical ward, procedures and processes) was assessed	(From former paper [33]) [31]	Y
	Audit and provide feedback	Monthly reports on clinician’s transfusion practices	[28]	Y
		Provision of real-time physician feedback	[23]	Y
	Conduct cyclical small tests of change	Two physicians from the medical group reviewed all messages generated by the system for 4 months prior to implementation and suggested modifications directed at ensuring that messages would be perceived as necessary, useful and brief	[26]	Y
	Develop a formal implementation blueprint	Both hospitals had a systematic approach for the implementation of computerised provider order entry (CPOE)	(From former paper [33]) [31]	Y
	Develop and organise quality monitoring system	At the end of PEWS implementation, compliance with PEWS performance and documentation was 100%	(From former paper [32]) [24]	N
		Review of noncompliant cases from the clinical data abstraction	[23]	Y
	Purposely re-examine the implementation	The implementation team evaluated the implementation process in a session at each ward with physicians and nurses	(From former paper [33]) [31]	Y
		Nurse educator: responsible for monitoring of PEWS quality	[24]	Y
	Stage implementation scale-up	A commercial patient data management system (PDMS) was introduced stepwise in each ICU subarea to replace the former paper-based patient chart	[25]	Y
		After a successful pilot, the PEWS system was implemented unit by unit in all non-paediatric ICU inpatient areas over a 6-month period	(From former paper [32]) [24]	N
		CPOE/CDSS was not simultaneously implemented in all study wards	(From former paper [33]) [31]	Y
		CPOE system was implemented incrementally over 2 years	[27]	Y
		Rollout from 2005 to 2011	[29]	Y
Provide interactive assistance	Centralise technical assistance	A member of the electronic patient record (EPR) staff was responsible for implementing it on the EPR system	[28]	Y
		Centralised IT staff required to maintain the PDMS	[25]	N
		Annual ongoing costs included help desk support	[30]	Y
		Ongoing help desk support. The help desk was staffed by one technical person and two clinical pharmacists who specialised in CPOE implementation	[27]	Y
	Provide clinical supervision	Full-time sepsis programme coordinator.	[23]	Y
		Nurse educator: responsible for on-the-ground support	[24]	Y
		Transfusion practitioner (TP) feeds back monthly reports on clinician’s transfusion practices in team meetings where open discussion is promoted to share experiences and as part of the education aspect of the meeting	[28]	Y
	Provide local technical assistance	During the actual implementation, the implementation team was available to answer questions and solve problems	(From former paper [33]) [31]	Y
Train & educate stakeholders	Conduct education meetings	Feedback reports on clinician’s transfusion practices discussed at monthly team meetings with education provided	[28]	Y
	Conduct ongoing training	Annual clinical personnel training	[29]	Y
		Conduct ongoing MD training & nursing skills fair	[23]	Y
		Personnel costs reflect … training	[27]	Y
		Training of nurses: initial 30-min training workshop & retraining exercises (6-h total) Nurse educator: responsible for PEWS training	[24]	Y
		Training of pharmacists and nurses	[30]	Y
		Training of physicians	[30]	N
		Training of physicians	[24]	N
		Training provided by TP: 30-min training sessions for new junior doctors on how to use the system	[28]	Y
		Physicians and nurses were introduced to and trained in the use of the system	(From former paper [33]) [31]	Y
	Develop educational materials	Develop physician/nurse training material	[23]	Y
		Training materials	[24]	Y
	Distribute educational materials	Pocket sepsis reference cards for nurses and doctors at the ED department	[23]	Y
	Make training dynamic	This introduction was different in both hospitals: demonstrations in one (passive learning) versus real practicing in prescribing (active learning) in the other	(From former paper [33]) [31]	Y
	Use train-the-trainer strategies	Building a clinical support team (train the trainer)	[30]	Y
		Training may have a ripple effect	[28]	N
Develop stakeholder interrelationships	Identify and prepare champions	Nurse educator	[24]	Y
		Physician champion	[30]	Y
		Use of TP as a champion	[28]	Y
	Involve executive boards	Executive committee to implement policies and procedures set forth by the steering committee	[23]	Y
	Model and simulate change	The modified PEWS was first piloted in the intermediate care unit at UNOP. During the pilot, we focused on identifying problems with the tool and making the necessary adjustments	(From former paper [32]) [24]	N
		Personnel costs: testing	[27]	Y
		Testing of the electronic medication management system (eMMS)	[29]	Y
	Use advisory boards and workgroups	A multidisciplinary team implemented a modified PEWS	[24]	N
		Sepsis steering committee: reviews sepsis cases and sends out timely weekly feedback, reviews and approves physician and nursing educational material and works with epic IT team to create new navigators, warning systems and order sets to improve recognition and management of sepsis	[23]	Y
	Use an implementation advisor	Research personnel (assuming researcher is an implementation scientist)	[23]	N
Adapt & tailor to the context	Promote adaptability	Involving physicians, pharmacists and nurses in the revision process.	[26]	Y
		Configuration of commercial CDSS (MedChart)	[29]	Y
		Modified the PEWS tool and algorithm from Boston Children’s Hospital for use at UNOP	(From former paper [32]) [24]	N
		Technical adjustments were made	(From former paper [33]) [31]	Y
		TP was involved in the design of the CDSS	[28]	Y
	Tailor strategies	Once the fully revised system was ready to go live, a memo was sent to the medical group’s providers to inform them of the new messages they would be receiving. The group had a history of including locally developed alerts and messages within their electronic medical records system so no further training was necessary	[26]	Y
Support clinicians	Create new clinical teams	The implementation process was performed by an implementation team consisting of information and communication technology and hospital pharmacy staff	(From former paper [33]) [31]	Y
	Facilitate relay of clinical data to providers	Monthly reports on clinician’s transfusion practices	[28]	Y
		Provision of real-time physician feedback	[23]	Y
Utilise financial strategies	Access new funding	Investment costs are covered by a hospital-wide PDMS implementation budget and derived from a separate PDMS investment plan for university hospitals with funding from the German government	[25]	N
		Financial incentives from the Centers for Medicare & Medicaid Services to providers who demonstrate meaningful use. At the top of the list of stage 1, meaningful use criteria are implementation of the CPOE system	[27]	Y
	Alter incentive/allowance structures	When the Everett Clinic meets prespecified quality benchmarking criteria, the pay-for-performance incentives are awarded annually by the health plans with which it contracts	[27]	Y
Change infrastructure	Mandate change	Executive committee to implement policies and procedures set forth by the steering committee	[23]	Y
		The boards of directors of both hospitals enforced their medical wards to implement CPOE	(From former paper [33]) [31]	Y
Other	Workflow alterations	Before the implementation, the current situation (organizational aspects of the medical ward, procedures and processes) was assessed	(From former paper [33]) [31]	Y
		Executive committee to implement policies and procedures set forth by the steering committee	[23]	Y
		Prescribing at the point of care demanded a fundamental shift in workflow	[27]	N
		Implementation also involved modification of nursing flowsheets to allow PEWS documentation and PEWS colour coding (green, yellow or red) on all unit census boards	(From former paper [32]) [24]	N
		Updating of hospital protocols and guidelines	[29]	Y
		Workflow-related issues	[30]	N

The Expert Recommendations for Implementing Change (ERIC) framework [19] was applied to categorise the implementation strategies. *N*, implementation strategy was not costed; *Y*, implementation strategy was costed

#### Use of evaluative and iterative strategies

Eight papers reported ‘using evaluative and iterative strategies’ [23–29, 31]. The most common implementation strategy in this cluster was *stage implementation scale-up* which was conducted in five of the papers [24, 25, 27, 29, 31] and costed in four papers [25, 27, 29, 31]. Stage up ranged from 6 months [24] to 6 years [29]. *Audit and provide feedback* [23, 28] and *purposely re-examine the implementation* [24, 31] were implementation strategies present in more than one included paper and consistently costed. Agulnik et al. were the only paper to partially cost the strategies reported under this cluster: the *purposeful re-examination of implementation* was costed, but the paper did not report costs for the three other strategies (the *assessment for readiness and identification of barriers and facilitators*, *staging implementation scale-up* and *development and organised quality monitoring system*) [24].

#### Provide interactive assistance

Seven papers ‘provided interactive assistance’ [23–25, 27, 28, 30, 31]. *Centralise technical assistance* was the most common implementation strategy in this cluster and was costed in all papers [27, 28, 30], except one [25]. Centralised technical assistance included centralised IT staff to maintain the CDSS [25], help desk support [27, 30] and "a member of the [electronic patient record (EPR)] staff was responsible for implementing it on the EPR system" [28]. Three papers reported *providing clinical supervision*, and all papers provided costs for the strategy [23, 24, 28].

#### Train and educate stakeholders

Seven papers reported strategies in the ‘train and educate stakeholders’ cluster, and all seven papers conducted *ongoing training* as an implementation strategy [23, 24, 27–31]. Training included: nursing skills fair [23], 30-min training workshop [24, 28], and annual clinical personnel training [29]. Three papers did not provide details of the training [27, 30, 31]. Most papers costed *ongoing training* except for two papers that partially costed it by reporting the costs for some clinicians (including pharmacists, nurses) but not for physicians [24, 30]. The final paper that partially costed implementation strategies reported under this cluster was Swart et al.; *ongoing training* and *educational meetings* were costed, but *train the trainer* was not [28]. *Develop educational materials* was present in more than one included paper and was consistently costed [23, 24].

#### Develop stakeholder interrelationships

Six papers ‘developed stakeholder interrelationships’ by employing five different implementation strategies [23, 24, 27–30]. *Identifying and preparing champions* was a common strategy in this cluster and was consistently costed across the papers [24, 28, 30]. Champions and the cost allocations included the followng: a nurse educator with 50% salary support [24], a transfusion practitioner at 0.8 full-time equivalent (FTE) [28] and a physician champion with an unclear cost allocation [30]. Only two papers partially costed this cluster [23, 24]. Afshar et al. costed two strategies (*use advisory boards and workgroups* and *involve executive boards*) but did not cost the final strategy (*use an implementation advisor*) in this cluster [23]. As mentioned, Agulnik et al. costed *identifying and preparing champions* but did not cost two other strategies: *model and simulate change* and *advisory boards and workgroups* [24].

#### Adapt and tailor to context

Five papers *promoted adaptability* of the CDSS and consulted clinicians in the process [24, 26, 28, 29, 31]. Clinician consultation occurred regardless if the CDSS was commercially sought, "configuration…required the equivalent of one full-time pharmacist" [29], or built in-house, "the Transfusion Practitioner was also involved in the design of the CDSS" [28]. One of the five papers also *tailored strategies* by leveraging an identified facilitator to substitute training for an informational memo sent to the competent clinician group [26]. Only one implementation strategy was not costed in this cluster [24].

#### Other implementation strategies

Three papers ‘supported clinicians’ by *facilitating the relay of clinical data to providers* [23, 28] and *creating new clinical teams* [31]. All implementation strategies in the cluster to support clinicians were costed [23, 28, 31]. Two papers reported implementation strategies under the cluster ‘utilise financial strategies’, but only one costed the strategies [25, 27]. Two papers described *mandating change* under the 'change infrastructure' cluster, and both papers costed this implementation strategy [23, 31]. None of the included studies reported any implementation strategies categorised in the ‘engage consumers’ cluster.

Six of the nine studies mentioned that workflows or protocols required alterations to assist with the uptake of CDSS [23, 24, 27, 29–31]. This implementation activity had no associated code under the ERIC framework; thus, the category ‘workflow alterations’ was created. One study explained that implementing the CPOE required a fundamental shift in workflow to allow, "prescribing at the point of care … and required a computer to be installed in each examination room" [27]. Prior to this CPOE implementation, prescribing occurred in offices or at workstations away from the patients [34]. One study did not expand on the extent of workflow alteration but mentioned "workflow-related issues" [30]. Three papers costed this implementation strategy [23, 29, 31].

### Measuring CDSS implementation costs

Table 3 outlines the approaches used by each study to measure and value implementation costs categorised as either ‘humans’, ‘things’ or ‘space’.

**Table 3:** Description of approaches used to measure and value implementation costs within the included papers

Paper	Method (valuation)		
First author, year, (reference)	Human	Things	Space
**Afshar, 2019 [**23**]**	Unclear (Bureau of Labor Statistics)	Unclear (market price)	✗
**Agulnik, 2019 [**24**]**	Unclear (actual salary)	Unclear (market price)	✗
**Castellanos, 2013 [**25**]**	Unclear (not reported)	✗	✗
**Field, 2012 [**26**]**	Activity diaries (Bureau of Labor Statistics)	✗	✗
**Forrester, 2014 [**27**]**	Unclear (Bureau of Labor Statistics)	✗	✗
**Swart, 2020 [**28**]**	Unclear (actual salary)	✗	✗
**Vermeulen, 2014 [**31**]**	Unclear (actual salary)	✗	✗
**Westbrook, 2015 [**29**]**	Activity diaries, interviews (actual salary)	Estimated over time horizon (market price)	Rental opportunity cost (market price)
**Zimlichman, 2013 [**30**]**	Unclear (Bureau of Labor Statistics)	✗	✗

The humans, ‘things’ and space costing framework [20] was applied

#### Humans

All papers reported ‘humans’ to be an implementation cost. Two papers measured staff labour directly using activity diaries [26, 29], and one paper also interviewed staff [29]. The activity diary used by Field et al. was weekly reports of staff member’s time spent on a set of predefined activities [26]. Westbrook et al. used work diaries and clinical personnel training schedules as well as interviewing "hospital pharmacists and from clinical information by IT and hospital managers who were involved in eMMS implementation and maintenance" to confirm the accuracy of the data [29]. Although Westbrook et al. recorded human-related implementation costs, these costs were not incorporated into the economic evaluation model as the authors assumed that "staff time spent in attending eMMS training sessions was incorporated into their existing workloads as no new staff were employed to cover their time" [29]. The authors further explained that they used an incremental approach to costing by only accounting for additional staff time associated with the eMMS implementation [29].

The remaining seven papers did not clearly report the methods they used to cost ‘humans’ [23–25, 27, 28, 30, 31]. The amount of time for planned implementation strategies was applied in four papers to cost personnel time. However, the authors did not explore the possibility of variations to scheduled implementation strategies, for example the need for additional training sessions or meetings that ran over the allotted time [23, 24, 27, 28]. Agulnik et al. measured training costs, "using the mean base salary for nurses at [National Paediatric Oncology] multiplied by the amount of time required for individual training" [24], and Swart et al. measured ongoing personnel costs from the listed activities including training and meetings [28]. Similarly, three papers reported the FTE portion of staff contributing to implementation strategies, but how the authors determined the FTE quota was not clear [24, 28, 29]. For example, Swart et al. costed their transfusion practitioner who assisted with implementation strategies, "at 0.8 FTE as this was appropriate to the project at the time these data were recorded" [28]. Two papers did not report any information on how personnel implementation costs were measured [30, 31].

The included papers valued staff time in three different ways. Four papers obtained the actual staff salaries at their respective hospitals [24, 28, 29, 31]. Four papers, all from USA, valued staff salaries from average rates for the region or nationwide obtained from Bureau of Labor Statistics [23, 26, 27, 30]. Castellanos et al. study described the salary rates that were applied in the costing but did not mention where these rates were obtained [25].

#### Things and space

Three papers costed ‘things’ associated with implementation strategies [23, 24, 29]. ‘Things’ were classified as consumables or durable assets [20]. Two papers costed consumables associated with education or training activities including "pocket sepsis reference cards" [23] and "training materials and study supplies (including paper, copies, educational materials, computer, Internet)" [24]. It was unclear how the consumables were costed, but the sunk costs appear to be reported. Westbrook et al. costed durable assets including furniture and equipment for training purposes, as well as custom equipment to improve usability [29]. The durable assets were estimated over the time horizon using the unit cost, useful lifespan and amounts reported. All ‘things’ were valued at market price. Only one paper costed ‘space’ associated with the implementation effort. Westbrook et al. included the rental opportunity cost where training took place and measured it at the market price attributed over a 15-year time horizon [29].

### Quality assessment

We used the 2022 CHEERS checklist to assess the reporting quality of economic evaluations in the included studies [21]. The percentage of not reported applicable items ranged from 12 to 42% across the papers. The following items were generally reported well in the papers: setting and location, outcome selection, measurement and valuation, costs and resource measurement and valuation, model rationale and description, characterising heterogeneity and uncertainty and the abstract, introduction, main results and discussion. Common limitations across all papers included the absence of reporting a health economic analysis plan and including the approach and effect of engaging with patients and others affected by the study. Additional limitations included not identifying the study as an economic evaluation in the title, study population, comparators, perspective, time horizon and discount rate. Additional file 2 contains detailed scores for each included study.

## Discussion

This systematic review identified nine papers that reported costs of implementation strategies relating to the introduction of a CDSS in a hospital setting. The relatively low number of included papers demonstrates the lack of reporting on implementation costs in this field. Implementation strategies and costs detailed in the included papers spanned all clusters of implementation strategies as defined within the ERIC framework, except for ‘engage consumers’. However, the methods used to cost implementation strategies were not well reported. Labour was the main implementation cost reported across the papers, irrespective of implementation strategy or cluster.

An absence of implementation costs in economic evaluations is problematic as it can contribute to an underestimation of costs, falsely optimistic cost-effectiveness estimates and a disconnect between published evidence and public health decision-making [35]. Nonetheless, the limited reporting on CDSS implementation costs that we describe here is consistent with studies evaluating implementation of digital health technologies and clinical innovations more broadly [35]. A 2006 systematic review investigated the impact health information technologies have on healthcare quality, efficacy and costs and was unable to determine cost efficiency as only 3 of 54 included studies reported the implementation costs [36]. A 2016 systematic review that evaluated the application of economic analysis within the field of improvement and implementation science found that only six of the thirty included studies described implementation costs [37]. These costs included preparatory work, training, education and ongoing costs regarding care quality and outcomes. All costs and outcomes were evaluated retrospectively leading the authors to propose that prospective economic evaluations in implementation studies may not be conducted due to a lack of awareness, perceived lack of importance, political or social sensitivities related to digital health investments or simply that they are not reported in academic publications or potentially other public forums.

Another explanation for the lack of reporting on implementation costs may be due to the lack of capability among digital health implementation evaluators compounded by a lack of appropriate methodological direction and practical tools for use in prospective economic evaluations within this field [38, 39]. Approaches to costing implementation strategies are still emerging. Saldana et al. developed a cost-mapping tool called the cost of implementing new strategies (COINS) [40]. The tool records and maps costs and resources used in an implementation process to the Stages of Implementation of Completion (SIC) framework in a manner similar to the time-driven activity-based costing approach, an established business accounting method [41]. COINS can measure the direct costs and indirect costs (e.g. personnel effort) required throughout implementation stages [41]. Further work by Sohn et al. developed a conceptual framework for assessing implementation costs [35]. The framework classifies implementation costs by resource type, key activities, implementation stage (design, initiation and maintenance), site level (site specific vs ‘above service’ or ‘central’ costs) and as programmatic versus non-programmatic (research) to allow for generalisation to other settings and for key drivers of implementation costs to be identified. Another recent approach by Cidav et al. combines time-driven activity-based costing with an implementation science framework, by Proctor et al., typically used to identify, specify and report implementation strategies and evaluate implementation effectiveness [8]. This is a pragmatic method that outlines the names, actions, actors and temporality for each implementation strategy, determines the duration of each action and then assigns a dollar value to the resources that each action consumes. The resulting data shows how specific components of an implementation strategy influence its overall cost. Finally, two papers have recently been published describing key considerations when costing implementation [10, 42]. Gold and colleagues explained how cost can be included and measured in implementation studies by utilising traditional economic evaluations that compare costs and effectiveness of health interventions [42]. Eisman and colleagues outlined that economic information should be presented in clear and meaningful ways to different stakeholder groups throughout the implementation effort and provide recommendations for cost assessment activities [10]. Despite these recent advances, there are no currently available methods or tools designed for the unique context of digital health implementation.

The digital health setting contains a range of additional challenges when costing implementation. In the included studies, we observed that ‘intervention’ versus ‘implementation’ costs were not clearly defined and often study specific. This ambiguity makes costing implementation strategies challenging [27]. For example, software and hardware are integral to the digital health innovation itself and therefore would typically be considered an intervention cost. However, Westbrook et al. described the introduction of long-life battery laptops fixed to custom trolleys in response to specific hardware issues that were identified in focus groups during the 1-year evaluation [29, 43]. In this sense, the laptops could be considered an adaption strategy to support implementation and therefore be included as an implementation cost. A clear set of common definitions around implementation in these contexts would minimise confusion.

Challenges defining CDSS implementation costs in a generalisable way can also be compounded by complexity associated with background levels of organisational digital maturity. Among organisations with higher levels of digital maturity, existing enterprise architecture and related processes, including business as usual workforce capacity building activities, may impact measurement of resource use and costs required for the implementation of new CDSS initiatives. Evaluating the costs of implementing a similar CDSS initiative in an organisation with a lower-level digital maturity may require additional types and volumes of implementation activities to achieve the same level of CDSS effectiveness. This also highlights the challenge of dealing with sunk implementation costs (e.g. prior investments in lifespan-limited information technology implementations on which a new CDSS is dependent) and opportunity costs (e.g. labour time diverted away from productive clinical care activities to participate in CDSS-related education and training activities). These issues were largely not addressed with methodological rigour among the included studies.

### Implications for costing implementation strategies in digital innovation

The methods used to cost implementation in the included papers adopted an ‘accounting’ approach to estimating costs; these approaches involve a financial analysis of direct operating costs [20]. However, for economic evaluations to inform healthcare investment policy, consideration of whether equal or greater health gains could have been achieved elsewhere for the same levels of investment is required [20, 44]. This may include consideration of indirect costs such as lost productivity. This is important, as economic costs have been shown to be 4 to 15% greater than accounting costs [20]. None of the current approaches or tools available to cost implementation considers opportunity costs [8, 35, 40]; the importance of opportunity costs has been highlighted in recent contributions to the literature [10, 42]. While considering stakeholders with diverse perspectives and key cost components during implementation, Eisman et al. stated that in implementation efforts which largely involve the time of frontline staff, productivity is a trade-off that should be estimated as an opportunity cost [10]. Additionally, Gold and colleagues have outlined that opportunity costs are specific to the decision-maker and may change over the time horizon [42].

CDSS are often implemented to increase service productivity. However, the introduction of a new software or change to established workflows is often accompanied by a ‘learning curve’ effect, where productivity may decline in the short term as staff adapt to the change [45, 46]. In the case of clinicians, the learning curve can make it difficult to provide the same standard of care in the same amount of time. A separate study that investigated barriers to CDSS implementation outlined that because a clinician’s pay schedule is attached to the number of patients seen daily, the learning curve becames a barrier to implementation [47]. Additionally, a systematic review that investigated the perceived barriers and facilitators of electronic health records (EHRs) implementation also found that loss of clinical productivity was a significant barrier by physicians. Conversely, productivity was perceived as a facilitator in studies that explored health professionals, managers and patients perceptions and demonstrated that EHRs were seen as positively influencing workplace efficiency and communication [48]. Two papers included in our review mentioned a learning curve but did not attempt to assign a cost to this, because it was assumed to be incorporated into existing workloads [29] or was not present due to a 2-year incremental implementation approach [27].

This review emphasises the importance of ‘workflow alterations’ as an implementation strategy in digital health innovation. While this is not a strategy previously identified in the ERIC framework, a newly developed evaluation framework for EHR-integrated innovations emphasises the importance of having an innovation seamlessly integrate into existing clinical and information system workflows [12]. Additionally, workflow challenges have been cited as a barrier to EHR adoption [4]. The papers included in this review only mentioned alterations to clinical workflows and not to information system workflows. However, only two of the five papers that mentioned workflow alterations reported that their CDSS was integrated with an EHR system [23, 27], and only one paper interviewed IT staff [29]. Workflow challenges appear to be a factor contributing to the complexity of implementing technology and should be considered when adopting digital health innovations.

An additional indirect cost consideration within the field of digital health relates to the expected life cycle of technology. Technology hardware has a relatively limited lifespan, typically between 2 and 7 years depending on replacement practices [29]. Only two of the studies included within this review considered technology lifecycle costs. Westbrook et al. calculated the total cost of hardware using its cost per unit and a 3-year useful life, repeated over 15-year time horizon, as well as determining the annualised cost attributed to the cardiology ward using the proportion of the number of beds in the cardiology ward [29]. In the other study, the expected life of hardware was the same as the time horizon, 5 years, and no specific lifespan considerations were incorporated into the cost analysis [27].

Software can also have a limited life cycle and can become obsolete or be iterated over time. This has been identified as an issue when the developing evidence base for specific programmes, as subsequent software generations can become available prior to the completion of a traditional randomised controlled trial [49, 50]. Depending on the nature of hardware and software assets, the costs of de-implementation of technology may need to be considered within the context of economic evaluation. De-implementation is its own process, separate to implementation, with relevant behaviour change strategies incurring additional resource use and associated costs [51]. Cost is often used as a justification for de-implementation, but, similar to implementation, little research exists on the costs associated with de-implementation strategies [52]. None of the included papers considered de-implementation, despite one analysing a time horizon of 15 years [29].

### Limitations

The strength of our findings is limited by the quality of the included studies. The quality of reporting varied across the papers, and missing information was not uncommon. Implementation strategies were not always clearly or comprehensively reported, although this is a common limitation in studies reporting on complex behaviour change interventions [53, 54]. None of the included studies employed an implementation science theory, model or framework; however, this trend may change with the recent development of a framework that addresses the unique challenges of implementing digital health innovations. The nonadoption, abandonment, scale-up, spread and sustainability (NASSS) framework was developed to predict and evaluate the success of technology-supported health (and social) care programmes [55]. Finally, all data in the included papers were collected retrospectively. Prospective data collection about implementation may allow for more comprehensive and accurate costings in future studies.

## Conclusion

Few papers have reported the costs associated with the implementation of CDSS in hospitals. Where these costs have been reported, there have been inconsistencies in terminology and approaches, and the methods used to assign costs were generally not well reported. Future research is needed to establish consistent terminology and appropriate methods for estimating and reporting on implementation costs within the context of digital health. Specific areas of focus should include accounting for technology life cycles including de-implementation costs, as well as the workforce productivity impacts associated with adapting to new technologies or processes.

## Supplementary Information

**Additional file 1.** Systematic review search string for each data base.

**Additional file 2.** Assessment of included studies against the 2022 Consolidated Health Economic Evaluation Reporting Standards (CHEERS) checklist.

## Data Availability

The authors declare that the data supporting findings of this review are available within the paper.

## Abbreviations

CDSS: Computerised decision support system
CHEERS: Consolidated Health Economic Evaluation Reporting Standards
COINS: Cost of implementing new strategies
CPOE: Computerised provider order entry
EHR: Electronic health record
eMMS: Electronic medication management system
EPR: Electronic patient record
ERIC: Expert Recommendations for Implementing Change framework
FTE: Full-time equivalent
IT: Information technology
PICOS: Population, intervention, comparator, outcomes, study design
PRISMA: Preferred Reporting Items for Systematic Reviews and Meta-Analyses
