# Supplementary material for: Implementation costs of hospital-based computerised decision support systems: a systematic review
Source: Implement Sci. 2023 Feb 24;18:7. doi: 10.1186/s13012-023-01261-8 (PMC9960445; doi:10.1186/s13012-023-01261-8)
Supplement: Supplementary file 1 — Additional file 1. Systematic review search string for each data base. [file 13012_2023_1261_MOESM1_ESM.pdf]

**Additional file 1:** Systematic review search string for each database.

| Database | PubMed | Embase | CINAHL | Scopus |
|---|---|---|---|---|
| **Search String** | ("Costs and cost analysis"[MeSH Terms] OR "cost*"[All Fields])<br><br>AND ("Implementation Science"[MeSH Terms] OR "implement*"[TIAB] OR "roll out"[TIAB] OR "scale up"[TIAB] OR "adopt*"[TIAB] OR "embed*"[TIAB] OR "integrat*"[TIAB])<br><br>AND ("Decision Support Systems, Clinical"[MeSH Terms] OR "Medical Records Systems, Computerized"[Mesh] OR "computerised clinical decision support system*"[TIAB] OR "artificial intelligence*"[TIAB] OR "machine learning*"[TIAB] OR "clinical information system*"[TIAB] OR "health information*"[TIAB] OR "early warning system*"[TIAB] OR "reminder system*"[TIAB] OR "dashboard*"[TIAB] OR "clinical decision support*"[TIAB])<br><br>AND ("Hospitals"[MeSH Terms] OR "hospital*"[TIAB] OR "medical cent*"[TIAB] OR "ward*"[TIAB] OR "emergency department*"[TIAB] OR "intensive care"[TIAB] OR "critical care"[TIAB] OR "outpatient*"[TIAB] OR "inpatient*"[TIAB] OR "secondary care"[TIAB] OR "tertiary care"[TIAB] OR "acute care"[TIAB ]) | ('health economics'/exp OR 'cost*')<br><br>AND ('implementation science'/exp OR 'implement*':ab,ti OR 'roll out':ab,ti OR 'scale up':ab,ti OR 'adopt*':ab,ti OR 'embed*':ab,ti OR 'integrat*':ab,ti)<br><br>AND ('clinical decision support system'/exp OR 'computeri$ed clinical decision support system*':ab,ti OR 'artificial intelligence*':ab,ti OR 'machine learning*':ab,ti OR 'clinical information system*':ab,ti OR 'health information*':ab,ti OR 'early warning system*':ab,ti OR 'reminder system*':ab,ti OR 'dashboard*':ab,ti OR 'clinical decision support*':ab,ti)<br><br>AND ('hospital'/exp OR 'hospital':ab,ti OR 'medical cent*':ab,ti OR 'ward*':ab,ti OR 'emergency department*':ab,ti OR 'intensive care':ab,ti OR 'critical care':ab,ti OR 'outpatient*':ab,ti OR 'inpatient*':ab,ti OR 'secondary care':ab,ti OR 'tertiary care':ab,ti OR 'acute care':ab,ti)<br><br>AND [2010-2021]/py<br><br>AND [embase]/lim<br><br>AND 'article':it<br><br>AND 'journal':pt | ((MH "Costs and Cost Analysis+") OR TX ("cost*"))<br><br>AND (TI ( "implement*" OR "roll out" OR "scale up" OR "adopt*" OR "embed*" OR "integrat*" ) OR AB ( "implement*" OR "roll out" OR "scale up" OR "adopt*" OR "embed*" OR "integrat*" ))<br><br>AND ((MH "Health Information Systems+") OR TI ( "computeri#ed clinical decision support system*" OR "artificial intelligence*" OR "machine learning*" OR "clinical information system*" OR "health information*" OR "early warning system*" OR "reminder system*" OR "dashboard*" OR "clinical decision support*" ) OR AB ( "computeri#ed clinical decision support system*" OR "artificial intelligence*" OR "machine learning*" OR "clinical information system*" OR "health information*" OR "early warning system*" OR "reminder system*" OR "dashboard*" OR "clinical decision support*" ))<br><br>AND ((MH "Hospitals+") OR TI ('hospital*' OR 'medical cent*' OR 'ward*' OR 'emergency department*' OR 'intensive care' OR 'critical care' OR 'outpatient*' OR 'inpatient*' OR 'secondary care' OR 'tertiary care' OR 'acute care') OR AB ('hospital*' OR | ALL ( "cost*" )<br><br>AND TITLE-ABS-KEY ( ( "implement*" OR "roll out" OR "scale up" OR "adopt*" OR "embed*" OR "integrat*" )<br><br>AND ( "computeri* clinical decision support system*" OR "artificial intelligence*" OR "machine learning*" OR "clinical information system*" OR "health information*" OR "early warning system*" OR "reminder system*" OR "dashboard*" OR "clinical decision support*" )<br><br>AND ( "hospital*" OR "medical cent*" OR "ward*" OR "emergency department*" OR "intensive care" OR "critical care" OR "outpatient*" OR "inpatient*" OR "secondary care" OR "tertiary care" OR "acute care" ) )<br><br>AND ( PUBYEAR > 2010 )<br><br>AND NOT INDEX ( medline )<br><br>AND ( LIMIT-TO ( DOCTYPE , "ar" ) )<br><br>AND ( LIMIT-TO ( SRCTYPE , "j" ) ) |

| | | | | |
|---|---|---|---|---|
| | AND (2010:2021[pdat])<br><br>AND "Journal Article"[pt]<br><br>NOT "Review"[pt]<br><br>NOT "Systematic Review"[pt] | | 'medical cent*' OR 'ward*' OR 'emergency department*' OR 'intensive care' OR 'critical care' OR 'outpatient*' OR 'inpatient*' OR 'secondary care' OR 'tertiary care' OR 'acute care'))<br><br>AND (PY 2010-2021)<br><br>AND (MX N)<br><br>AND (PT Journal Article) | |
| **Number of results** | 901 | 437 | 409 | 634 |
