# Supplementary material for: Implementation costs of hospital-based computerised decision support systems: a systematic review
Source: Implement Sci. 2023 Feb 24;18:7. doi: 10.1186/s13012-023-01261-8 (PMC9960445; doi:10.1186/s13012-023-01261-8)
Supplement: Supplementary file 2 — Additional file 2. Assessment of included studies against the 2022 Consolidated Health Economic Evaluation Reporting Standards (CHEERS) checklist. [file 13012_2023_1261_MOESM2_ESM.pdf]

Additional file 2: Assessment of included studies against the 2022 Consolidated Health Economic Evaluation Reporting Standards (CHEERS) checklist.

Item No.	Afshar, 2019	Agulnik, 2019	Castellanos, 2013	Field, 2012	Forrester, 2014	Swart, 2020	Vermeulen, 2014	Westbrook, 2015	Zimlichman, 2013
**1**	Yes	Yes	No	No	Yes	No	Yes	Yes	No
**2**	Yes	Yes	Yes	Yes	Yes	Yes	Yes	Yes	Yes
**3**	Yes	Yes	Yes	Yes	Yes	Yes	Yes	Yes	Yes
**4**	No	No	No	No	No	No	No	No	No
**5**	Yes	Yes	No	Yes	Yes	No	Yes	Yes	No
**6**	Yes	Yes	Yes	Yes	Yes	Yes	Yes	Yes	Yes
**7**	No	Yes	Yes	No	Yes	Yes	Yes	Yes	No
**8**	Yes	No	No	No	Yes	Yes	Yes	Yes	No
**9**	Yes	No	Yes	No	Yes	Yes	Yes	Yes	Yes
**10**	No	No	No	No	Yes	No	Yes	Yes	Yes
**11**	Yes	Yes	Yes	Yes	Yes	Yes	Yes	Yes	Yes

**12**	Yes	Yes	Yes	Yes	Yes	Yes	Yes	Yes	Yes
**13**	Yes	Yes	Yes	Yes	Yes	Yes	Yes	Yes	Yes
**14**	Yes	Yes	Yes	Yes	Yes	Yes	Yes	Yes	Yes
**15**	Yes	Yes	Yes	No	Yes	Yes	Yes	Yes	Yes
**16**	Yes	Yes	n/a	n/a	Yes	Yes	Yes	Yes	Yes
**17**	Yes	Yes	n/a	n/a	Yes	Yes	Yes	Yes	No
**18**	Yes	n/a	n/a	n/a	n/a	n/a	n/a	n/a	n/a
**19**	Yes	No	n/a	n/a	Yes	n/a	n/a	n/a	n/a
**20**	Yes	Yes	n/a	n/a	Yes	Yes	Yes	Yes	Yes
**21**	No	No	No	Yes	No	No	No	No	No
**22**	Yes	Yes	Yes	Yes	Yes	Yes	Yes	Yes	No
**23**	Yes	Yes	Yes	Yes	Yes	Yes	Yes	Yes	Yes
**24**	Yes	Yes	No	No	Yes	Yes	Yes	Yes	Yes
**25**	No	No	No	No	No	No	No	No	No

**26**	Yes	Yes	Yes	Yes	Yes	Yes	Yes	Yes	Yes
**27**	Yes	Yes	Yes	Yes	Yes	No	Yes	Yes	No
**28**	Yes	Yes	Yes	Yes	No	Yes	Yes	Yes	No
**% not reported** $= \boldsymbol{No/(n/a - 28)}$	18%	26%	35%	39%	15%	27%	12%	12%	42%
